# Radio Frequency IDentification for Meat Supply-Chain Digitalisation

**DOI:** 10.3390/s20174957

**Published:** 2020-09-01

**Authors:** Paolo Barge, Alessandro Biglia, Lorenzo Comba, Davide Ricauda Aimonino, Cristina Tortia, Paolo Gay

**Affiliations:** 1 Department of Agricultural, Forest and Food Sciences (DiSAFA), Università degli Studi di Torino, Largo Paolo Braccini 2, 10095 Grugliasco (TO), Italy; paolo.barge@unito.it (P.B.); alessandro.biglia@unito.it (A.B.); lorenzo.comba@unito.it (L.C.); davide.ricauda@unito.it (D.R.A.); paolo.gay@unito.it (P.G.); 2 CNR-IEIIT—Politecnico di Torino, Corso Duca degli Abruzzi 24, 10129 Torino, Italy

**Keywords:** UHF and HF RFID, NFC, food industry, meat traceability, blockchain, supply-chain

## Abstract

Digitalised supply-chain traceability systems can offer wide prospects both for improving safety as well as enhancing perceived quality. However, the coupling between physical goods and information is often difficult for agri-food items. A solution could be the use of RFID (Radio Frequency IDentification) systems. Due to its wide reading range, Ultra-High Frequency (UHF) technology is already widely used in logistics and warehousing, mostly for the identification of batches of items. A growing interest is also emerging in Near Field Communication (NFC), as several smartphones embed an integrated NFC antenna. This paper deals with the automatic identification of meat products at item level, proposing and evaluating the adoption of different RFID technologies. Different UHF and NFC solutions are proposed, which benchmark tag performances in different configurations, including four meat types (fatty beef, lean beef, chicken and pork), by using a specifically designed test bench. As avoiding the application of two different tags could be advantageous, dual frequency devices (UHF and NFC) are also considered. Significant differences in tag performances, which also depend on meat type and packaging, are highlighted. The paper highlights that tag positioning should consider the geometry of the packaging and the relative positioning of tag, meat and reader antenna.

## 1. Introduction

It is well recognised that, especially for meat products, high consumer trust and the perceived good reputation of the food influence the customer’s buying experience, resulting in higher sales. Apart from mandatory traceability, which is governed by specific national laws, voluntary traceability is often adopted, with the goal being to share additional food attributes or more detailed traceability information that could reach the consumer through other information media. This is particularly attractive for new marketing strategies [1].

Since meat traceability was introduced early in Europe to tackle the spread of diseases, mandatory data are collected in huge official databases (e.g., animal registries and sanitary databases), which contain the main traceability information of each single head (birth, age, transport, slaughter etc.). In Italy, the consumer is allowed to access part of this information (birth date, sex, breed, breeding farm code and slaughtering date) on the national registry website by typing the animal’s 14-letter code [2].

Labelling rules are very strict. National and supranational beef producers, together with food authorities, often debate about the possibility of allowing the implementation of additional information on labels or sharing it by other means of communication (e.g., apps, websites).

**Figure 1:**
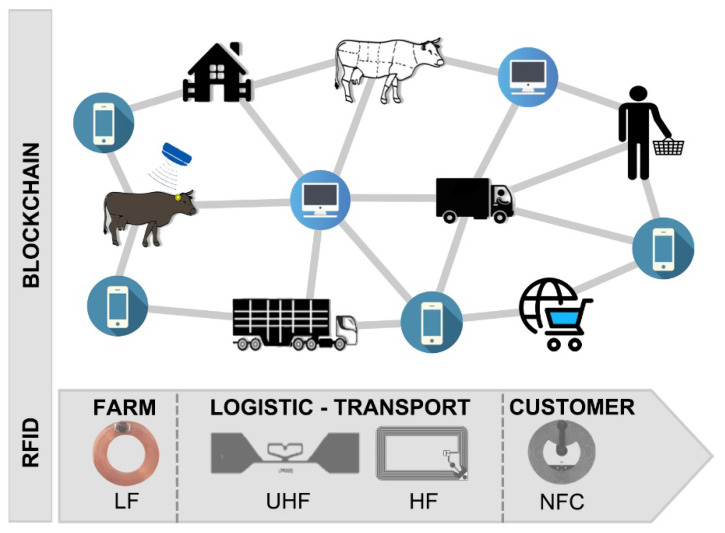
Example of meat supply-chain digitalisation. The Low Frequency (LF) ear-tag contains the animal code which is registered in private and public repositories. High Frequency (HF) and Ultra-High Frequency (UHF) tags identify meat cuts, and Near Field Communication (NFC) tags are used on packed meat in retail. The distributed and decentralised data infrastructure using blockchain enhances the level of trust in the traceability system.

High quality meat producers could increase their income through a private certified information flux, which is costly to maintain nowadays, subject to a low degree of identification system standardisation and dependent on certification of trustworthiness by third parties. This situation causes the information flux on meat supply-chain traceability to be discontinuous and very costly, excluding a priori a great part of the possible stakeholders. To enhance the reliability of supply-chain traceability and its robustness against possible external inferences, the information about meat origin and processing could be guaranteed by blockchain technologies [3,4,5]. The disintermediation offered by blockchain technologies could solve part of the problems tied to the efforts of guaranteeing a trustworthy, efficient traceability system without the involvement of subjects who are external to the supply chain and who act as a trusted third party. Nevertheless, the physical identification of a single food item, packed or bulk, is often critical. Radio Frequency IDentification (RFID) technologies are also gaining attention in the field of intelligent meat packaging for accurate, real-time traceability and automatic sorting systems [6,7,8], which could be authenticated in joined RFID-blockchain systems [9,10]. Secure individual item tracking and secure data product association could be valuable methods in fighting counterfeits. In the meat supply chain, different types of tags for automatic identification, paired to blockchain authentication, can be used; an example of integration of RF technologies in a digitalised meat supply-chain traceability system is shown in Figure 1.

Ultra-High Frequency (UHF) RFID technologies have been proposed for the food sector, e.g., fresh produce [11,12], cold-chain logistics [13,14] and the beverage industry [15]. Most of these studies have been focused on the identification of batches of products contained in boxes, crates, pallets or containers, which are identified by UHF tags, leading to traceability systems based on larger units [1,16]. In many applications for food delivering and handling, radio-frequency meat traceability technologies were applied positioning the tag on secondary packaging [17], while item-level UHF RFID identification of meat cuts has not been reported in the literature. Badia-Melis et al. [18] evidenced the potential benefits of introducing RFID (UHF paired to Near Field Communication (NFC)) for the traceability of the whole food chain until the consumer. An RFID-based traceability network was modelled and implemented in a beef supply chain in China from animal husbandry to meat distribution, involving the use of UHF RFID labels [19]. Nonetheless, technical issues associated with UHF labelling on single meat cuts were not discussed. Furthermore, a UHF RFID system was proposed for item-level identification of fish [20].

Meat supply-chain traceability is indeed complicated due to the huge amount of data required by regulations, by information privacy levels (also involving health issues) and by the willingness of the stakeholder to transmit/acquire certain information that cannot be directly perceived by the senses (credence attributes). In Europe, the information that can be reported on printed labels is strictly regulated by Regulation (EU) No 1169/2011 [21] and other specific laws. Therefore, in many cases, producers are not able to transmit information related to topics, normally voluntarily traced, that are very valuable to buyers. Meat supply-chain digitalisation should serve to guarantee both the compliance with mandatory requirements as well as a more flexible interactive approach in the meat information flow.

Electronic animal identification is currently mandatory for sheep and goats and well regulated for cattle in the European Union. It is allowed to be performed with Low Frequency (LF) ISO 11784 and 11785 standard passive tags [22,23], which report the animal’s unique ID stored in the National Animal Registry. In the slaughter process, which is very critical as for other bulk products [1,24], High Frequency (HF) or Ultra-High Frequency (UHF) tags can be directly applied to the meat on the carcass or on the meat cuts to guarantee traceability during chilling and transport [25] (Figure 1).

While Electronic Product Code (EPC) communication standards in the UHF or HF band can be exploited for processing and logistics traceability, the end user could benefit from Near Field Communication (NFC) technology by using new generation mobile phones [26]. Nowadays, NFC tags are proposed for various applications such as payment, where the buyer scans the NFC tag on the product and then proceeds to pay electronically while skipping the cashier line; in the future, this could be an alternative to the traditional way of buying. Information about discounts, loyalty marketing, recommendations, product history, composition and recipes could also be made available during shopping, thus enhancing consumer experience.

The labels, which are normally placed on the plastic wrapping, always report the mandatory animal data. For example, in Europe, it is strictly required to report animal code, birth date, birth country, slaughter plant code and slaughter plant country. Voluntary traceability data could be added without further requirements, e.g., age at slaughter, sex, meat category, breeding period in the country and breeding region. In Italy, other traceability and quality data (e.g., feed, breeding system, sanitary treatments and welfare strategies) can be added on the packaging only if appropriately certified and under the strict control of the Ministry of Agriculture. Then, pictures and other marketing/selling information could be printed.

An increasing quantity of fresh meat is sold cut and packed and is displayed on the shelves of self-service refrigerated cabinets. The most frequent type of meat packaging displayed in supermarkets consists of a tray overwrapped with plastic film or in vacuum packaging. In the first case, air (or a modified atmosphere) surrounds the meat cut, while in vacuum packaging the air is removed by negative pressure in sealed pouches. Therefore, the distance between the labels and the meat varies from approximately 100 microns (the thickness of a plastic film for vacuum pouches) to a few centimetres. While RFID technologies have been proposed and used in the food supply chain for some commodities, UHF RF identification of meat cuts at item level is not yet available.

It is known that animal tissues and food composition could negatively affect high-frequency electromagnetic signal propagation [27,28,29]. Meat itself is very problematic in RF identification as it contains a large amount of water, which causes attenuation and degradation of the electromagnetic signal. While the reliability of RF identification is known to be potentially critical for some food products [30,31], there is a lack of available data in the literature about tag performance when attached to meat.

This paper deals with the problem of the RFID identification of processed meat products (e.g., hamburgers) at item level, which is required in retail. With the aim of defining the possible criticalities of any stakeholder in meat cut identification, RFID system frameworks were envisaged to perform item-level identification at different points of the meat supply chain. In particular, systems operating in the UHF frequency band were considered in the cutting plants and refrigeration phase where item identification can be performed by fixed panels as well as by mobile Personal Digital Assistants (PDAs) similar to those already used in other warehousing applications, while NFC systems were considered for products to be detected on the shelves by user mobile phones. Dual frequency tags, already used in different sectors [32,33,34] make it possible to exploit the advantages of both UHF and NFC.

This paper analyses the criticalities in packed meat food RF identification and proposes possible solutions to enhance the reliability of RFID system integration for meat logistics and traceability based on experimental indices that are related to readability. The overall performances of the RF systems were tested on four different kinds of packed minced meat (fatty beef, lean beef, pork and chicken), by using two available RFID technologies (UHF or HF-NFC) and mixed-mode tags (UHF and HF-NFC) in order to avoid the use of two different identifiers.

A series of experimental trials to evaluate the feasibility of automatic meat RF identification was conducted by considering two different meat packaging options: a) shrink bags or vacuum bags, with the film in direct contact or very close to the meat, and b) overwrapped trays, with the film applied to the tray, with a modified atmosphere or air between the film and the meat. With this aim in mind, two different test bench layouts were designed for UHF and HF readability testing, respectively. Dual Frequency (DF) tags were tested on both test benches to assess their performances in comparison with the Single Frequency (SF) tags.

The paper is organised as follows: Section 2 introduces the UHF and NFC test benches used for the experiments, the adopted tags, the preparation and composition of the meat samples, and the statistical methodology adopted in the paper. Section 3 reports the results of the experimentation which are discussed in Section 4. Finally, in Section 5, conclusions are drawn.

## 2. Materials and Methods

### 2.1. Description of the UHF Test Bench

**Figure 2:**
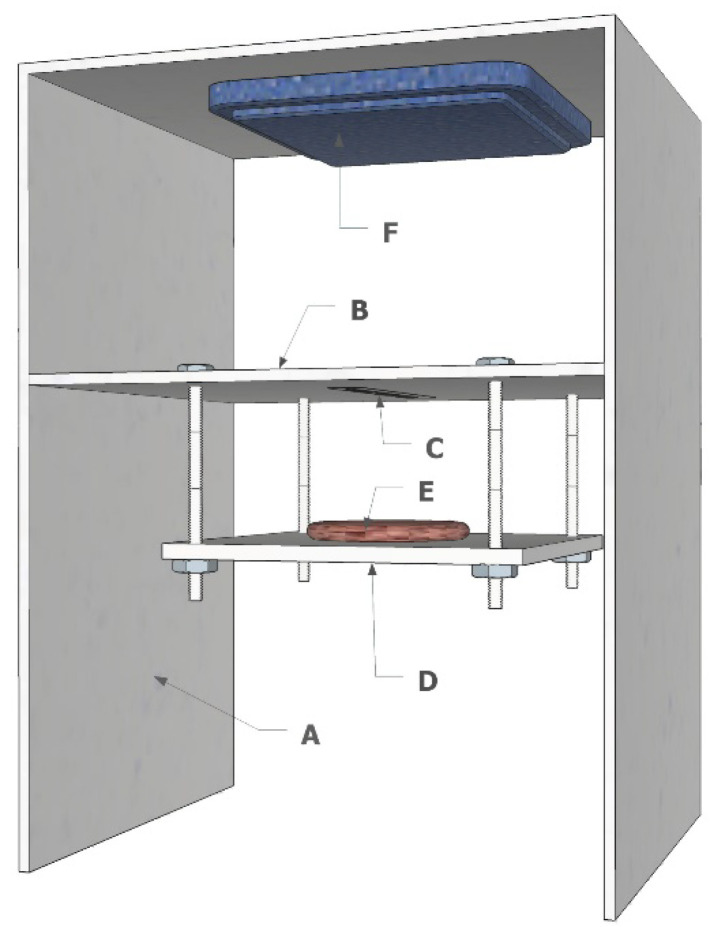
UHF test bench: (**A**) low-density polystyrene box, (**B**) central fixed shelf, (**C**) UHF tag, (**D**) adjustable platform, (**E**) meat sample and (**F**) UHF panel antenna.

The three-dimensional layout of the designed test bench for the UHF RFID system performance assessment is shown in Figure 2. The main structure of the test bench was a box (Figure 2A) equipped with a fixed central shelf (Figure 2B) where the tag was attached (Figure 2C). An adjustable platform (Figure 2D), hosting the meat samples (Figure 2E), was hung from the fixed shelf. The box, the shelf and the platform were made of low-density polystyrene. Polystyrene as well as other types of plastic were used as their low dielectric constant and loss factor minimally affect the performance of UHF antennas [27]. The system makes it possible to set the tag-to-meat distance, in the range 0–18.75 mm, by fastening a polyethylene screw (0.625 mm per each step). The UHF panel antenna (8 dBi gain, linear polarisation, manufactured by Caen RFID, Viareggio, Italy, model Wantenna X007) (Figure 2F) was attached to the ceiling of the polystyrene box, fixing the tag-to-antenna distance at 0.5 m. The tag antenna was perpendicular to the direction in which the wave was moving, centred to the reader antenna. The tag antenna was optimally oriented to linear field polarisation of the reader antenna. A commercial standalone reader was used, operating at 866 MHz (Ion 4301P, Caen RFID, Viareggio, Italy, EPC Class-1 Gen-2 compliant) and connected to the polarised linear antenna. The reader was connected to the antenna by means of a 5 m length coaxial cable type Amphenol TFC/l U-JIN, model type TWB 1951 (0.365 dB/m attenuation at 20 °C) and two connectors (TNC female and N-type male), which further caused a total 0.36 dB attenuation.

To determine the reading efficiency of the system, the minimum Transmitted Power Output (TPO) required to activate the tag (*P*_min_ (dBm)) was evaluated by a custom C# software, [31], which controlled the reader, generated an increasing RF-transmitted power output ramp in the range 0–33 dBm and automatically switched off as soon as the tag was detected and the EPC code correctly acquired. The experiment started with the tag in contact with the meat sample, to simulate meat in vacuum packaging. To study the case of the tray overwrapped with plastic film, the tag-to-meat distance was increased, obtaining fixed distance values at 0.625 mm steps in the range 0–18.75 mm. Three repetitions were performed for each tag-to-meat distance. For each meat type, the tested tag order was randomly scheduled to avoid any influence due to the different test duration.

### 2.2. Description of the HF Test Bench

**Figure 3:**
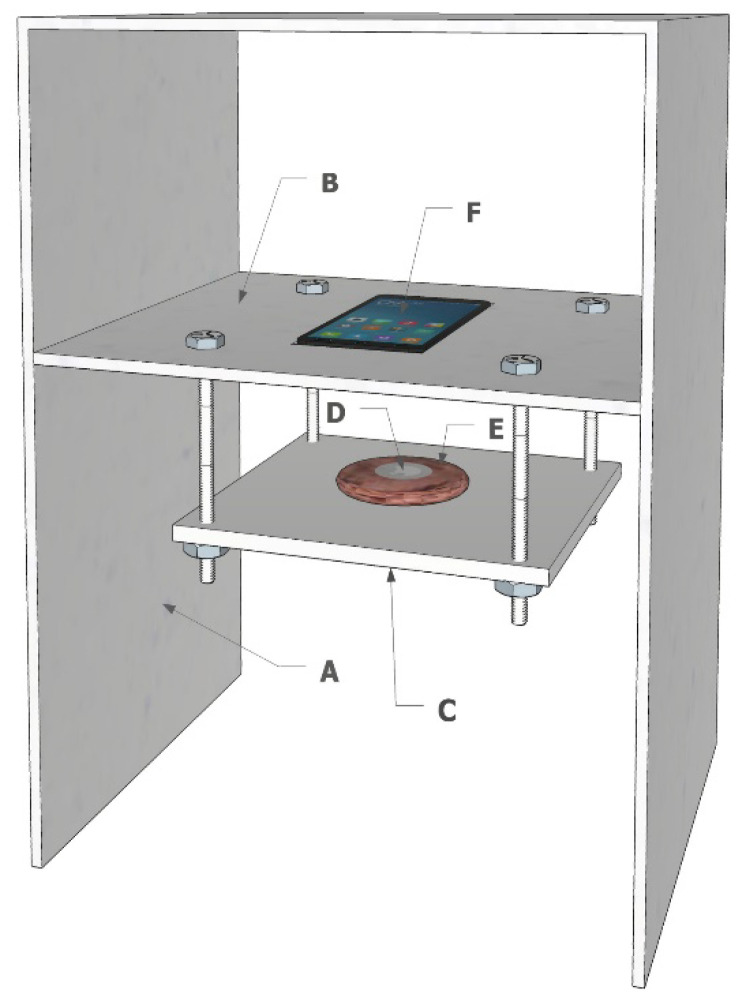
HF test bench: (**A**) low-density polystyrene structure, (**B**) central fixed shelf, (**C**) adjustable platform, (**D**) NFC tag, (**E**) meat sample and (**F**) Android smartphone embedding the NFC reader.

Figure 3 shows the test bench layout for the HF RFID system performance assessment. The HF test bench structure was similar to the one used for the UHF tests (Figure 2), as the low-density polystyrene box, the fixed central shelf and the adjustable platform were maintained (Figure 3A–C). The reading system, a commercial smartphone, was leaned against the fixed shelf. The HF tag was laid in direct contact with the meat sample in a central position (Figure 3D–E) to simulate the most critical position for tag readability. A test was performed without the meat sample to simulate packaging where the meat is laid in trays with overwrapped film.

In this trial, two different Android smartphones (Samsung Galaxy S8 and Xiaomi Mi 8 models; Figure 3F), embedding the NFC reader, were used to perform tag detection. Samsung and Xiaomi smartphones were chosen as they have been listed in the top five best-selling smartphone brands since 2018. Apple smartphone models were not tested as in the iOS system, the NFC features are reserved for Apple Pay applications. On both tested smartphones, a free Android application (NXP Taginfo by NXP Semiconductors) for the reading/writing of NFC tags was installed. Since the NFC antenna positions of the tested smartphones are different (in the Samsung Galaxy the antenna is approximatively in the centre of the phone, while in the Xiaomi it is mounted in the upper part, near the camera), the meat sample was shifted to align the centres of the reader and tag antennas, based on the technical sheet of the phone.

In the initial test configuration, the tag-to-smartphone distance was set to 100 mm in order to keep the tag outside the smartphone reading range. Then, the NXP Taginfo application was activated and set to continuous tag reading. The tag-to-smartphone distance was then progressively decreased by turning the screw system in 0.625 mm steps. When detection was obtained, the tag-to-smartphone distance (*R*_max_ (mm)) was measured and recorded. Three repetitions were performed for each tag-meat-smartphone combination. For each meat type, the tested tag order was randomly scheduled to avoid any influence due to the different test duration.

### 2.3. Description of the Tested Tags

The features of the tags used in the experiment are reported in Table 1, Table 2 and Table 3. Table 1 reports the tested UHF tags. Due to their overall performance and long reading range, they are commonly used in logistics. The chip embedded in the UHF tags is the Impinj Monza 5 or NXP Semiconductors Ucode 7, which presents a higher reading sensitivity. All the UHF tags are compliant with the ISO/IEC 18000-63 and EPC Gen2 V2 standards.

The dual band tags (Table 2) are equipped with the EM4423 integrated circuit, which is the latest generation of EM microelectronic contactless devices. It combines two functionalities: (1) the EPC technology that is used for long-range applications, and (2) the NFC technology that is used to exchange data at proximity range. The EM4423 chip supports the ISO/IEC 14443 Type A, NFC Forum Type 2 specifications, as well as the ISO/IEC 18000-63 and EPC Gen2 V2 protocols. The chip comes with a 96-bit read-only identifier that is shared in both RF protocols. Tag 2.A (Table 2) has the same UHF antenna as tag 1.C. The three inlays were chosen for their shapes and dimensions, which can be adapted to different types of packaging. The dual band tags were tested by using both the UHF as well as the HF test benches.

The NFC tags (Table 3) are compliant with the ISO 14443/IEC Type A and NFC Forum 2 international standard; they are equipped with a re-writable user memory. Tags 3.A and 3.B (Table 3) have a novel tamper evidence function for the identification of unauthorised access and the manipulation of a product, package or system.

Tampering attempts are detected and permanently stored in the chip’s memory. Tags 3.C–3.H in Table 3 are commonly used in general NFC applications due to their performance, suitable form-factor and cost. Tags 3.C and 3.E have the same inlay, but tag 3.C has a bigger memory size, is attached to a film, and is commercialised as a high-performance tag designed for the identification of metal objects. The inlay of tag 3.F is similar to the one of tags 3.C and 3.E, but it has a smaller size.

### 2.4. Meat Sample Preparation and Chemical Characterisation

The meat samples were prepared by grinding 100 g of lean beef, fatty beef, pork or chicken meat and reshaping to obtain 100 mm diameter patties by using a metal ring. The meat samples were pressed softly with a plastic disc to minimise as much as possible the presence of air inside the minced meat. The height of the patties was approximatively 10 mm, depending on the meat type.

Since some chemical compounds (water, salts etc.) drastically influence tag readability, especially when the tag and the sample are very close [15,31], the meat samples were analysed to evaluate the main chemical features that can influence tag readability: dry matter (AOAC method 950.46 [35]), crude proteins (AOAC method 928.08 [35]), ether extract (AOAC method 991.36 [35]) and ashes (weight after heating at 550 °C for 5 h). Table 4 reports the results of the chemical analysis. Water content proved to be very similar for lean beef, pork and chicken, while it was slightly lower for fatty beef. Chicken and lean beef contained higher amounts of both ashes and proteins than fat beef and pork. Fatty beef and pork contained more fat than lean beef and chicken, with lean beef having the lowest amount of fat.

The experimental tests using the designed test benches were conducted with samples refrigerated at approximatively 4 °C to simulate the product temperature of chilled retail cabinets. It was therefore necessary to periodically cool the samples before each measurement.

### 2.5. Statistical Analysis

The obtained *P*_min_, acquired by means of the UHF test bench, was considered as the dependent variable and analysed by a linear model to evaluate the effects of the considered factors: meat type and tag model (as categorical variables) as well as the distance between the tag and meat surface (as the numerical regressor). Two-fold and three-fold interactions were considered in the model. In the case of the NFC tags, a three-way ANOVA (ANalysis Of VAriance) was used, evaluating the effects of the meat, tag and smartphone model categorical variables on the dependent variable *R*_max_. The mean values of the dependent variables (*P*_min_ and *R*_max_) were evaluated by using the post-hoc Bonferroni test for *p* < 0.01. The R software (version 3.6.3) was used to process the data.

## 3. Results

### 3.1. Effects of Meat Proximity on EPC UHF Tag Detection

An index accounting for the UHF tag’s performance, in terms of tag readability when attached to a food item, was obtained by measuring the amount of power needed during the interrogation phase in order to guarantee a feasible response from the tag at a given distance.

**Figure 4:**
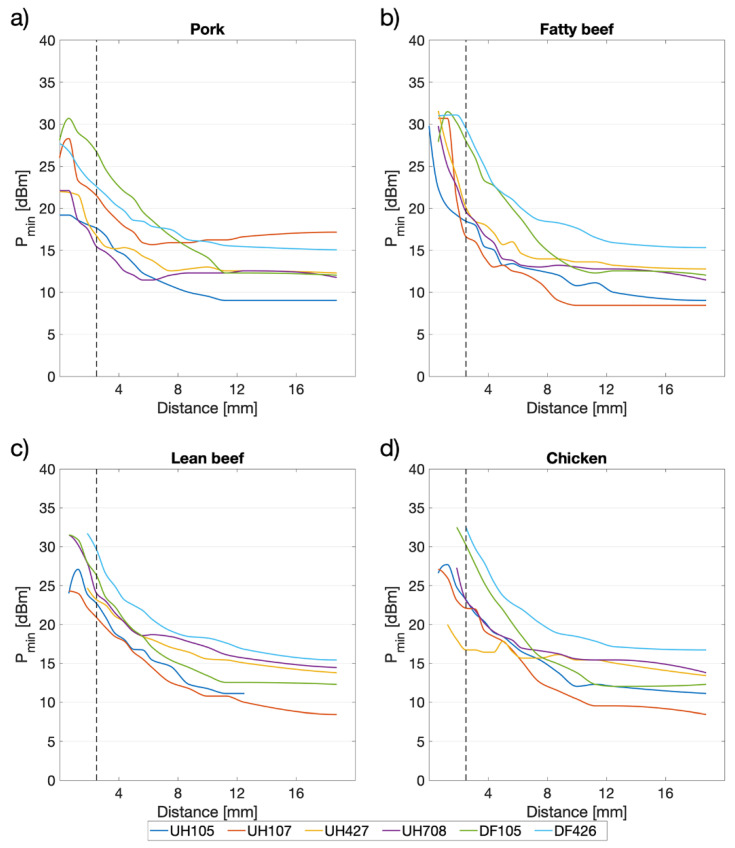
Mean values of the minimum power required to activate the UHF and DF tags (*P*_min_ (dBm)) at increasing tag-to-meat distances (mm) when tested on different types of minced meat patties. The dashed lines highlight the minimum distance at which all the six tags were detected. Subfigure (**a**) regards pork, Subfigure (**b**) regards fatty beef, Subfigure (**c**) regards lean beef, while Subfigure (**d**) regards chicken.

In the carried-out experiments, the effects of meat proximity on tag performance were clearly observable. In particular, when the tag was in contact or very close (<2.5 mm) to the meat (Figure 4), the level of power required to obtain a tag response was high. In these conditions, some tags were not even detected. Nevertheless, all the tested tags turned out to be readable at a tag-to-meat distance greater than 2.5 mm for each type of meat (indicated by the dashed lines in Figure 4). Table 5 reports, for all tags, the minimum tag-to-meat distance needed to obtain at least one detection and successful reading of the tag’s EPC code out of three repetitions.

It can be noticed that contact with pork meat affects tag performance minimally, as in most cases the tags were also readable when they were laid directly on the patty’s surface (Table 5). In the other cases, except for two tags in contact with fatty and lean beef, a certain distance had to be maintained to obtain tag activation. The minimum reading distances vary considering the different meat and tag types.

To assess significant differences in tag performance, in terms of the minimum amount of power to be delivered to activate the tags, *P*_min_ values at a distance in the range 2.50–18.75 mm were compared statistically. Values at shorter distances (<2.5 mm) were not compared as many tags were not readable in this interval. In the linear model, the meat type, tag and distance factors were considered in all combinations, in order to evaluate their effects on the dependent variable *P*_min_. The variance was explained by all of the three considered factors and factor interactions (coefficient of determination R^2^ = 0.999). The *P*_min_ means by tag factor, grouped by means of the Bonferroni test at *p* < 0.01, are reported in Table 6.

**Figure 5:**
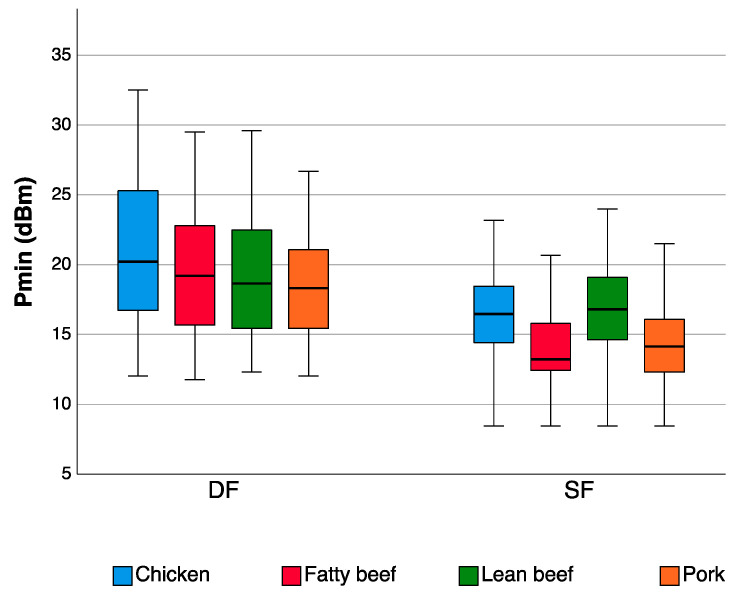
Comparison of the Single Frequency (SF) and Dual Frequency (DF) tag types on four meat types when detection is performed in the UHF band. The data are expressed as the minimum power required to activate the tags (*P*_min_ (dBm)) on a box-plot graph. The tag types are compared in the 2.5–18.75 mm range of distances.

Tags UH 105 and UH 107, whose inlay shapes are very similar, proved to perform very well on the meat for the low power rates required for their detection. Compared to the Single Frequency tags, the activation of the Dual Frequency tags was possible only by emitting higher energy levels (Table 6, Figure 5).

The *P*_min_ means by meat factor, grouped by means of the Bonferroni test at *p* < 0.01, are reported in Table 7. The effect of the meat type factor on the UHF tag’s performance was significant (*p* < 0.01). The readability of the tag was significantly higher in proximity to pork or fatty beef, rather than chicken or lean beef.

In Figure 4, the *P*_min_ mean values are reported for each meat type in the 0–18.75 mm distance range. It should be noted that, when the tag and the meat were almost in contact (<2.5 mm), tag readability was severely compromised. Indeed, the *P*_min_ values were very high or even out of the reader’s TPO range (>33 dBm). Table 8 reports the *P*_min_ mean values at the different tag-to-meat distances considering all the tags and all the meat types.

However, in most cases, *P*_min_ constantly decreases as a function of the tag-to-meat distance (Figure 4 and Table 8). In Figure 5, the two types of tags (Single Frequency and Dual Frequency) were compared in the 2.5–18.75 mm range of distances. It was noticed that Single Frequency tags were much more efficient than Dual Frequency tags as the *P*_min_ values were lower.

### 3.2. Effects of Meat Proximity on NFC HF Tag Detection

The maximum scanning distance measured between the reading smartphone and the tags attached to the different meat patties (*R*_max_) turned out to be in the range 15–51.25 mm. The meat type factor did not have a statistically significant effect on the variable *R*_max_.

The maximum tag-to-smartphone reading distance was significantly lower when using the Samsung smartphone rather than the Xiaomi model (Table 9). The smartphone model factor was significant for *p* < 0.01 by using the Bonferroni test.

Regarding the NFC tags, the shape and size of the antenna loop heavily influenced successful tag detection. Larger tags (e.g., circular tag 3.D and tag 3.G, Table 3) were more easily detectable at longer distances.

With Dual Frequency tags, readability is possibly influenced more by the size of the HF antenna loop rather than by the technology (Single or Dual Frequency) employed. In fact, DF tags with larger HF antenna loops (2.A and 2.C, Table 10) had readabilities similar to other medium-sized single-frequency tags. The reading distance of Dual Frequency tag 2.B (Table 10) proved to be comparable to Single Frequency tag 3.H, whose HF antenna loop size was similar. The Dual Frequency tags were easily readable in terms of distance but it was observed that, in comparison with the Single Frequency tags, the time needed to receive the EPC tag code was at least doubled.

The anti-metal film, which was attached to tag 3.C (Table 10) to improve the tag’s performance when applied to the metal, had a negative effect on the tag’s maximum reading distance when the tag was in contact with the meat sample. In fact, the maximum reading distance of tag 3.C was lower when compared to tag 3.E (Table 10), which has the same inlay and chip.

## 4. Discussion

RFID can be used in advanced meat packaging of fresh meat cuts of different animal origins and compositions, allowing item-level contactless identification both in the UHF as well as in the HF (NFC) frequency bands. In this paper, the effects of meat proximity on the readability of RFID systems were assessed. Due to the high amount of energy required to activate the tag, low reading performances arose in the UHF frequency band when the tags were placed in contact with or very close to the meat (Figure 4 and Table 6). In some tag/meat type combinations, the transponders were not even readable in the distance range 0–2.5 mm (Table 5). This could be an issue for some types of packaging (e.g., vacuum sealed packages), where the places available to attach RFID tags are limited. When the tag was attached to the meat product, the technical characteristics of the tag antenna were affected and changed depending on the dielectric properties of the meat [36]. In particular, the meat mass changed the overall impedance of the antenna, degrading the tuning efficiency of coupling between the antenna and the tag integrated circuit, and absorbing the electromagnetic signal [28].

UHF tags require a certain amount of signal power to be activated and transmit the Electronic Product Code (EPC), which is related to the item’s traceability information. As shown by Barge et al. [31], who considered tag readability when tags are attached to packed liquid products, the presence of high amounts of fat, which has a low dielectric constant, impacted positively on UHF tag readability. Accordingly, the research showed that the tags in the vicinity of pork and fatty beef (Figure 4a,b), which are fattier than chicken and lean meat (Figure 4c,d), were detected even when the tag was closer to the meat patty surface.

The use of samples of the same weight and shape made it possible to compare the effects of the meat type on tag readability. Among other factors, differences due to the meat type and the tags were assessed in controlled static conditions, which allowed the variability caused by the speed or by the EPC anti-collision algorithm to be reduced.

High values were found in terms of the power needed for tag activation, also considering that, in experimental conditions, the distance between the item and the reader antenna was only 0.5 m, while, in some logistics applications, the distance might be larger. This could be the case in RFID gates or conveyor belts for multiple dynamic identification; in this context, the reading performance of the UHF system might be further reduced.

In some cases (e.g., tag UH 105 in Figure 4c,d), the minimum amount of power for tag activation when very close to the meat (distance < 0.625 mm) was lower than the value obtained at the subsequent distance step. This could be due to the combined contribution of different phenomena that can alter the electromagnetic tag/reader coupling, such as the absorption of electromagnetic waves by the meat mass and the detuning effect on the tag antenna lying on the meat surface.

Mc Carthy et al. (2010) [36], who considered only beef meat muscle in their experiments (1 and 2 kg), reported that dynamic identification can also be affected by meat proximity. According to the results presented in their study, Mc Carthy et al. (2010) also found that tag type significantly influenced reading performances.

Based on the reported results, some precautions will have to be adopted to improve tag readability, such as adopting plastic or carton trays that allow the meat parts to be maintained at a suitable distance from the tag. This can be obtained, for example, considering an appropriate distance between tag and meat as well as mutual orientation of the transponder with the reader antenna. In fact, especially in the case of chicken and lean beef, positioning the tag in proximity to the meat should be avoided, choosing instead, for example, a packaging solution where a layer of another material or atmosphere is interposed between the RFID identifier and the product.

Due to the possibility of setting the distance between the tag and the meat sample, the designed UHF test bench allowed the effects of meat sample proximity on tag readability to be clearly highlighted. The designed HF tag test bench allowed the maximum reading distance between NFC tags attached to the meat patty surface and the smartphone to be defined.

Further work should focus on the effects of different orientations and distances in UHF identification by means of circular and/or linear antennas that can be mounted at strategic points where identification is considered in warehousing and at points of sale (shelves, machines that handle food in warehousing) or used by operators (by means of PDAs).

NFC tags were shown to be suitable for the scenario where the consumer uses a smartphone app to obtain information to access the digitalised supply chain. The large-scale use of tags could enhance product sales as shoppers, during their buying, are time-pressed; however, an eventual failure of the identification system could be fatal if smartphones are sought to influence the choice of picking one product over another. As different smartphone models determined significant differences in the maximum reading distances, further testing on more recent smartphone models could be performed in future research. The difference between the reading performances of the two smartphone models can probably be ascribed to better radio interfaces and/or different NFC antenna designs [32].

The availability of Dual Frequency tags could be a good opportunity to use RFID both in logistics as well as for other proximity range applications such as at points of sale. From user-level access to the digital dataflow through a trusted identification system, each stakeholder can benefit from the given information. In particular, some non-perceivable quality attributes (e.g., production methods, animal breeds, farm locations, sustainability, requirements related to ethnic and religious groups, details on ethics and many others) could be guaranteed by a complete digitalisation of the meat supply chain. By means of smart technologies, the consumer is involved in an in-store selling engagement, enjoying a service that is similar to that offered by e-commerce, and post-buying access to the information they might not have enough time to consider while shopping. Moreover, retailers can have the advantage of tracing locations of customers during their buying path, cross-selling some suggested items based on marketing strategies.

## 5. Conclusions

The adoption of UHF tags in the animal-origin supply chain has been considered for many years both for food [37,38,39] as well as for cattle [40,41,42,43]. However, RFID UHF technology is not yet widespread in the food and breeding sectors. The reason for this is that there are still some technological problems that prevent its reliability and therefore its usage. The deployment of RFID in meat supply-chain digitalisation would allow both more efficient management of meat processing and warehousing data flows as well as an improved consumer purchasing experience.

In this paper, the weaknesses and advantages of using UHF and/or NFC systems to perform automatic identifications, which could be adopted in logistics as well as at points of sale, were assessed. The research showed that there are configurations in which tag reading performance would result to be too low to be applicable in real contexts. The distance between the transponder and the reader antenna resulted to be a critical parameter for a good performance of the UHF RFID system. The dimensions of the air gap that should be maintained were assessed and reported.

For NFC applications, the effect of the distance between the tag and the meat as well as the performance of recent smartphone models could be further investigated.

It was demonstrated that, in many cases, reliability problems in reading performances can be overcome by carefully choosing the tag model and its positioning. Tags could be integrated into food packaging, developing suitable solutions that can guarantee the relative positioning of the meat and the transponder during the handling of the product throughout the entire supply chain. When properly integrated, UHF technology can thus lead to successful applications. The results reported in this paper can be exploited by developers of sensors based on RFID technology for meat applications.

Further experimentation should also assess the effects of other meat cuts or meat products (e.g., offal, marrow) with different dielectric properties.

## Figures and Tables

**Table 1:** Technical specifications of the Ultra-High Frequency tags used on the test bench in Figure 2.

No.	Tag Inlay	Integrated Circuit	Chip Nominal Reading Sensitivity (dBm)	Antenna Size Width × Length (mm)	Inlay Shape ^1^
1.A	Lab ID UH 105	Impinj Monza 5	−20	91 × 18	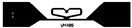
1.B	Lab ID UH 107	NXP UCODE 7	−23	91 × 18	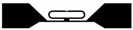
1.C	Lab ID UH 427	NXP UCODE 7	−23	50 × 30	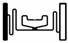
1.D	Lab ID UH 708	NXP UCODE 7	−23	70 × 12	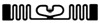

^1^ Tag pictures are not reported in real dimensions and scale.

**Table 2:** Technical specifications of the Dual Frequency tags used on both test benches in Figure 2 and Figure 3.

No.	Tag Model	Integrated Circuit	Antenna Size Width × Length (mm)	Inlay Shape ^1^
2.A	DF 426	EM Marin 4423	HF: 30 × 15 UHF: 50 × 30	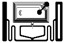
2.B	SMARTRAC WEB DF	EM Marin 4423	HF: 19 × 11 UHF: 50 × 30	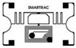
2.C	DF 105	EM Marin 4423	HF: 30 × 15 UHF: 84 × 27.7	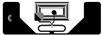

^1^ Tag pictures are not reported in real dimensions and scale.

**Table 3:** Technical specifications of Near Field Communication tags tested on the test bench in Figure 3.

No.	Tag Model	Integrated Circuit	Antenna Size Width × Length (mm)	Inlay Shape ^1^
3.A	Tamper Loop Circus™	NXP NTAG 213	∅ 19 total length 54 *19*	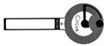
3.B	NT4H2421 Tx Tamper	NXP NTAG 213	∅ 20 total length 35	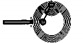
3.C	Anti-metal NTAG	NXP NTAG 216	∅ 25	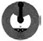
3.D	Smartrac bullseye™	NXP NTAG 213	∅ 35	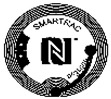
3.E	Mini tag	NXP NTAG 213	∅ 25	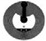
3.F	NTAG	NXP NTAG 213	∅ 20	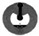
3.G	NTAG-X	NXP NTAG 213	20 × 35	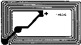
3.H	Midas tag NFC	NXP NTAG 213	10 × 17	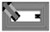

^1^ Tag pictures are not reported in real dimensions and scale.

**Table 4:** Chemical composition of the meat samples.

Meat Type	Water % Content	Ashes % of Dry Matter	Crude Protein % of Dry Matter	Total Fat % of Dry Matter
Fatty beef	70.87	3.38	76.41	14.26
Pork	73.16	3.81	75.57	13.31
Chicken	73.90	4.75	85.44	7.06
Lean beef	73.52	4.32	85.98	4.30

**Table 5:** Minimum tag-to-meat distances (mm) required to read each UHF tag, considering the Single or Dual Frequency tag model and the four meat types.

No.	Tag Model	Type ^1^	Pork	Fatty Beef	Lean Beef	Chicken	Mean
1.A	UH105	SF	0.00	0.00	1.25	0.625	0.47
1.B	UH107	SF	0.00	1.25	0.00	0.625	0.47
2.C	DF105	DF	0.00	0.625	0.625	1.875	0.78
1.C	UH427	SF	1.25	0.625	1.875	1.25	1.25
1.D	UH708	SF	0.00	1.25	1.875	1.875	1.25
2.A	DF426	DF	0.00	1.875	1.875	2.50	1.56
	Mean		0.21	0.94	1.25	1.46	

^1^ SF = Single Frequency; DF = Dual Frequency.

**Table 6:** Tag factor effects. Mean values of the minimum power needed to activate the tag (*P*_min_ (dBm)) for each of the considered tags when in proximity to minced meat patties (in the 2.50–18.75 mm tag-to-meat distance range).

No.	Tag Model	Type	*P*_min_ (dBm) ^1^
1.B	UH 107	SF	14.6 ^a^
1.B	UH 105	SF	14.2 ^a^
1.C	UH 427	SF	16.0 ^b^
1.D	UH 708	SF	16.0 ^b^
2.C	DF 105	DF	18.4 ^c^
2.A	DF 426	DF	20.7 ^d^

^1^ Different letters (a, b, c, or d) indicate that the means are significantly different by using the Bonferroni test for *p* < 0.01.

**Table 7:** Meat type factor effects. The mean values of the minimum power necessary to activate the tag (*P*_min_ (dBm)) are reported for each of the considered meat types in the 2.50–18.75 mm tag-to-meat distance range.

Meat	*P*_min_ (dBm) ^1^
Pork	15.5 ^a^
Fatty beef	15.7 ^a^
Lean beef	17.5 ^b^
Chicken	17.9 ^b^

^1^ Different letters (a or b) indicate that the means are significantly different by using the Bonferroni test for *p* < 0.01.

**Table 8:** Tag-to-meat distance factor effects. The mean values of the minimum power needed to activate the tag (*P*_min_ (dBm)) are reported for each tag-to-meat distance in the range 2.50–18.75 mm.

Distance (mm)	*P*_min_ (dBm)
2.50	22.67
3.12	21.38
3.75	19.87
4.37	18.83
5.00	17.84
5.62	16.95
6.25	16.29
7.50	15.17
8.75	14.39
10.00	13.78
11.25	13.30
12.50	13.08
18.75	12.48

**Table 9:** Mean of the maximum tag-to-smartphone reading distance (*R*_max_ (mm)) with the two smartphones models.

	*R*_max_ (mm)			
Smartphone Model	Mean ^1^	STD dev	Min	Max
Samsung Galaxy S8	29.16 ^a^	8.41	15.00	45.00
Xiaomi Mi 8	36.00 ^b^	7.79	18.75	51.25
Total	32.58	8.77	15.00	51.25

^1^ Different letters (a or b) indicate that the means are significantly different by using the Bonferroni test for *p* < 0.01.

**Table 10:** Maximum scan distance (*R*_max_ (mm)) means of the NFC tags attached to a meat patty (mm). The data show the means for the two considered smartphone models.

No.	Tag	Type	*R*_max_ (mm) ^1^	STD dev (mm)
3.D	Smartrac bullseye™	SF	44.61 ^a^	4.13
3.G	NTAG-X	SF	42.37 ^a^	5.16
3.E	Mini tag	SF	36.87 ^b^	4.87
2.A	DF 426	DF	35.47 ^bc^	5.52
2.C	DF 105	DF	33.24 ^bc^	8.27
3.B	NT4H2421TxTamper	SF	32.24 ^bcd^	6.53
3.A	Tamper Loop Circus™	SF	30.87 ^cde^	6.68
3.F	NTAG	SF	28.24 ^def^	7.47
2.B	Smartrac web DF	DF	26.24 ^ef^	5.51
3.C	Anti-metal NTAG	SF	24.89 ^f^	5.24
3.H	Midas tag NFC	SF	23.37 ^f^	6.17

^1^ Different letters (a, b, c, d, e and f) indicate that the means are significantly different by using the Bonferroni test for *p* < 0.01.

## References

[1] Dabbene F., Gay P., Tortia C. (2014). Traceability issues in food supply chain management: A review. Biosyst. Eng..

[2] Italian Bovine Registry, Veterinary System Database. https://www.vetinfo.it.

[3] Azzi R., Kilany C.R., Sokhn M. (2019). The power of a blockchain-based supply chain. Comput. Ind. Eng..

[4] Westerkamp M., Victor F., Küpper A. (2020). Tracing manufacturing processes using blockchain-based token compositions. Digit. Commun. Netw..

[5] Lanko A., Vatin N., Kaklauskas A. Application of RFID combined with blockchain technology in logistics of construction materials. Proceedings of the International Science Conference SPbWOSCE-2017 “Business Technologies for Sustainable Urban Development”.

[6] Kerry J.P., O’Grady M.N., Hogan S.A. (2006). Past, current and potential utilization of active and intelligent packaging systems for meat and muscle-based products: A review. Meat Sci..

[7] McMillin K.W. (2017). Advancements in meat packaging. Meat Sci..

[8] Comba L., Belforte G., Gay P. (2013). Plant layout and pick-and-place strategies for improving performances in secondary packaging plants of food products. Packag. Technol. Sci..

[9] Tian F. An agri-food supply chain traceability system for China based on RFID & blockchain technology. Proceedings of the 13th International Conference on Services Systems and Services Management, ICSSSM.

[10] Cena F., Boella G., Cordero A., Guffanti A., Rapp A., Schifanella C., Gay P., Tortia C., Barge P., Comba L. Blockchain and Artificial Intelligence for quality food protection and advanced consumer services. Proceedings of the 5th Italian Conference on ICT for Smart Cities and Communities.

[11] Mainetti L., Mele F., Patrono L., Simone F., Stefanizzi M.L., Vergallo R. (2013). An RFID-based tracing and tracking system for the fresh vegetables supply chain. Int. J. Antennas Propag..

[12] Fan B., Qian J., Wu X., Du X., Li W., Ji Z., Xin X. (2018). Improving continuous traceability of food stuff by using barcode-RFID bidirectional transformation equipment: Two field experiments. Food Control.

[13] Jedermann R., Stein K., Becker M., Lang W. UHF-RFID in the Food Chain—From Identification to Smart Labels. Proceedings of the 3rd International Workshop.

[14] Laniel M., Émond J.-P., Altunbas A.E. (2011). Effects of antenna position on readability of RFID tags in a refrigerated sea container of frozen bread at 433 and 915 MHz. Transp. Res. Part C Emerg. Technol..

[15] Barge P., Biglia A., Comba L., Gay P., Ricauda Aimonino D., Tortia C. (2017). Temperature and position effect on readability of passive UHF RFID labels for beverage packaging. Chem, Eng. Trans..

[16] Karlsen K.M., Donnelly K.A.M., Olsen P. (2011). Granularity and its importance for traceability in a farmed salmon supply chain. J. Food Eng..

[17] Mc Carthy U., Ayalew G., Butler F., McDonnell K., Ward S. (2011). The case for UHF RFID application in the meat supply chain in the Irish context: A review perspective. Agric. Eng. Int. CIGR J..

[18] Badia-Melis R., Mishra P., Ruiz-García L. (2015). Food traceability: New trends and recent advances. A review. Food Control.

[19] Liang W., Cao J., Fan Y., Zhu K., Dai Q. (2015). Modeling and Implementation of Cattle/Beef Supply Chain Traceability Using a Distributed RFID-Based Framework in China. PLoS ONE.

[20] Trebar M., Lotrič M., Fonda I., Pleteršek A., Kovačič K. (2013). RFID data loggers in fish supply chain traceability. Int. J. Antennas Propag..

[21] (2011). Regulation (EU) No 1169/2011 of the European Parliament and of the Council of 25 October 2011 on the provision of food information to consumers. Off. J. Eur. Union.

[22] International Organization for Standardization (1996). ISO Standard 11785:1996: Radio Frequency Identification of Animals—Technical Concept.

[23] International Organization for Standardization (1996). ISO Standard 11784:1996 Radio Frequency Identification of Animals—Code Structure.

[24] Comba L., Belforte G., Dabbene F., Gay P. (2013). Methods for traceability in food production processes involving bulk products. Biosyst. Eng..

[25] Barge P., Gay P., Merlino V., Tortia C. (2013). RFID technologies for livestock management and meat supply chain traceability. Can. J. Anim. Sci..

[26] Pigini D., Conti M. (2017). NFC-Based Traceability in the Food Chain. Sustainability.

[27] Bolić M., Simplot-Ryl D., Stojmenović I. (2010). RFID Systems: Research Trends and Challenges.

[28] Dabbene F., Gay P., Tortia C., Iakovou E., Bochtis D., Vlachos D., Aidonis D. (2016). Chapter 8: Safety and Traceability. Supply Chain Management for Sustainable Food Networks.

[29] Barge P., Gay P., Merlino V., Tortia C. Effect of packaging on Radio Frequency IDentification of food products. Proceedings of the XXXV CIOSTA & CIGR Conference: From effective to intelligent agriculture and forestry.

[30] Mc Carthy U., Ayalew G., Butler F., Donnell K.M., Lyng J., Ward S. (2009). Permittivity of Meat Fish and their Components at UHF RFID Frequencies and Industry Relevant Temperatures. Agric. Eng. Int. CIGR J..

[31] Barge P., Biglia A., Comba L., Gay P., Ricauda Aimonino D., Tortia C. (2019). The influence of food composition and tag orientation on UHF RF IDentification. J. Food Eng..

[32] Rizzi A., Volpi A., Rinaldi R., Bandinelli R., Gonzalez A., Hardgrave B. (2019). Assessing the performances of RFID UHF and HF dual-frequency apparel tags. Int. J. RF Technol..

[33] Volpi A., Rizzi A., Rinaldi R., Rinaldi R., Bandinelli R. (2019). Dual Frequency Tag Performances in the Fashion Industry. Business Models and ICT Technologies for the Fashion Supply Chain.

[34] Mayer L.W., Scholtz A.L. Dual-band HF / UHF Antenna for RFID. Proceedings of the 68th IEEE Vehicular Technology Conference.

[35] AOAC International (2000). AOAC Official Methods of Analysis.

[36] Mc Carthy U., Ayalew G., Butler F., McDonnell K., Ward S. (2010). The effect of increased interrogation zone, reader antenna polarization and application factors in the performance of UHF RFID tag detection on modified atmosphere packaged meat. Packag. Technol. Sci..

[37] Barge P., Gay P., Merlino V., Tortia C. (2014). Item-level Radio-Frequency IDentification for the traceability of food products: Application on a dairy product. J. Food Eng..

[38] Dabbene F., Gay P., Tortia C. (2016). Radio-Frequency Identification Usage in Food Traceability. Advances in Food Traceability Techniques and Technologies: Improving Quality throughout the Food Chain.

[39] Barge P., Gay P., Merlino V., Tortia C. (2013). UHF-RFID solutions for logistics units management in the food supply chain. J. Agric. Eng..

[40] Hammer N., Adrion F., Staiger M., Holland E., Gallmann E., Jungbluth T. (2016). Comparison of different ultra-high-frequency transponder ear tags for simultaneous detection of cattle and pigs. Livest. Sci..

[41] Barge P., Gay P., Piccarolo P., Ricauda Aimonino D., Tortia C., Caria M., Chessa G., Murgia L., Pazzona A., Todde G. RFID identification and traceability of Agnello Sardo lamb. Proceedings of the XXXVI CIOSTA CIGR V Conference 2015 “Environmentally Friendly Agriculture and Forestry for Future Generations”.

[42] Umstätter C., Bhatti S.A., Michie C., Thomson S. Overview of Ultra-High Frequency technology in livestock farming and stakeholder opinions. Proceedings of the International Conference of Agricultural Engineering.

[43] Wisanmongkol J., Pongpaibool P. A passive UHF RFID tag for poultry traceability. Proceedings of the International Symposium on Antennas and Propagation (ISAP).

